# A Month for Gallbladder Cancer: A Case Report of Four Gallbladder Cancers Over a 30-Day Period at a Community Hospital

**DOI:** 10.14740/wjon951w

**Published:** 2015-12-31

**Authors:** William Patrick Boyan Jr., Stephen Klepner, Michael Farr, Adam Kopelan, Prakash Paragi, Kevin Clarke

**Affiliations:** a Monmouth Medical Center, Long Branch, NJ, USA; b Newark Beth Israel Medical Center, Newark, NJ, USA

**Keywords:** Gallbladder cancer, Radical cholecystectomy

## Abstract

Gallbladder carcinoma is an exceedingly rare and fatal cancer. With stage 1 disease having a 5-year survival of only 50%, a diagnosis of gallbladder cancer carries a grim prognosis. Fortunately, especially in the developed world, it is exceedingly rare. Annual rates are reported as low as 1 - 2.5 per 100,000 people in the United States. Over the past 14 years, Newark Beth Israel in New Jersey has diagnosed 21 patients with gallbladder cancer. The purpose of this case report was to describe four new cases which occurred over a 30-day period in 2015 and review the risk factors, diagnosis and treatment strategies for this terrible disease.

## Introduction

Gallbladder carcinoma is an exceedingly rare malignancy in the developed world. In the United States, it accounts for only 0.5% of all gastrointestinal (GI) cancers, making it the fifth most common GI cancer in the country. Globally, incidence rates vary widely reaching very high rates in North and South American women. According to one study in 2008, in the United States, there are less than 5,000 cases of gallbladder cancer reported annually (1 - 2.5 per 100,000) [1]. However, the American Cancer Society estimates that in 2015, there will be 10,910 new cases of gallbladder and other biliary cancers reported [2]. Unfortunately, due to its early vague symptoms and the gallbladder’s lack of serosa to slow its spread, gallbladder carcinoma typically presents at advanced stages and carries a 5-year survival rate of less than 5% [1].

Other than ethnicity and geographic location, case studies have elucidated other risk factors that may contribute to the pathogenesis of gallbladder cancer, mainly gallstones, chronic gallbladder inflammation and presence of polyps greater than 1 cm in size [1]. Cholelithiasis is found in approximately 85% of patients with gallbladder cancer. Larger stones (> 2 - 3 cm) carry a greater association with cancer [1]. This may help to explain the higher rates of malignancy in females; however, only 1-3% of patients with cholelithiasis develop gallbladder cancer [3]. Gallbladder cancer is found incidentally on histological examination in 0.3-3% of laparoscopic cholecystectomies performed for symptomatic cholelithiasis [1]. The remaining patients with gallbladder carcinoma present with symptoms secondary to advanced disease and carry a dismal prognosis.

The purpose of this case study was to describe four incidents of gallbladder carcinoma, which occurred over the course of 1 month at Newark Beth Israel Hospital in Newark, New Jersey.

## Case Report

An unusual occurrence of four cases of gallbladder cancer occurred at Newark Beth Israel medical center (NBI) over a 31-day span. As a reference, since 2001, NBI has had 21 cases of gallbladder cancer and the institution did 2,606 cholecystectomies between January 2003 and June 2015, averaging 208.5 operations per year over that time.

### Case 1

The first case was a 52-year-old female with a history of gastroesophageal reflux disease (GERD) and hypertension who presented with a 2-month history of abdominal pain and weight loss. An ultrasound of her gallbladder revealed only sludge. A prior esophagogastroduodenoscopy (EGD) showed biopsies positive for *H. pylori* and the patient was treated without resolution of symptoms. CT done by the emergency department showed a distended gallbladder with 1.2 cm mass. This patient underwent an exploratory laparotomy which showed diffuse peritoneal studding and bulky portal lymphadenopathy. A biopsy of the lymph node was taken and showed moderately differentiated adenocarcinoma. At this time, it was determined that doing a cholecystectomy would be very technically difficult and being that she was stage IV, the procedure would not benefit the patient oncologically. The patient was closed and will be undergoing chemotherapy.

### Case 2

The next patient was an 86-year-old female with a history of dementia, sick sinus syndrome with a pacer, stroke, diverticulitis, hypothyroidism, and diabetes. She presented with a 2-day history of right upper quadrant pain and was tender on exam. The patient’s WBC was 17.5 and her total bilirubin was 2 mg/dL. Ultrasound showed gallstones, fluid around the gallbladder, wall thickening and common bile duct (CBD) dilation. A CT was done in the emergency department which showed stones and fluid around the gallbladder. The patient was taken for laparoscopic cholecystectomy with intraoperative cholangiogram due to her pacemaker excluding MRCP. No filling defects were seen on cholangiogram and the gallbladder was extracted in an endoscopic retrieval bag. There was nothing grossly abnormal about the specimen. Pathology showed a 1 cm moderately differentiated adenocarcinoma with negative margins. This represented stage 2 (T2Nx) disease. After discussion with the family and primary physician, it was determined that due to this patient’s advanced age and comorbidities, she would undergo only chemotherapy.

### Case 3

A 72-year-old female with a history of hypertension presented with diffuse abdominal pain and decreased appetite for 1 day. On exam, she had right upper quadrant tenderness, rebound and a positive Murphy’s sign. Ultrasound showed stones, fluid around the gallbladder and wall thickening. CT showed wall thickening as well as pneumobilia. The patient’s white count was 16/μL and as there was concern for emphysematous cholecystitis, the patient was taken to the OR for laparoscopic cholecystectomy. Again the specimen appeared normal and was retrieved in a specimen bag. Pathology showed a stage 3b cancer with involvement of a lymph node from the cystic duct (T2N1). The patient is following up with the surgeon for liver resection, as well as oncology for chemotherapy plans.

### Case 4

The final case was a 74-year-old female with a history of hypertension, rheumatoid arthritis, GERD and diabetes. She came to the emergency with 1-day history of epigastric pain. A CT showed stones, fluid around her gallbladder, gallbladder wall thickening and a normal CBD of 6 mm. Ultrasound of the abdomen reported appearance of a 4 cm mass but could not rule out that it was stones and sludge. An ERCP was done which extracted sludge as well as an endoscopic ultrasound which showed gallbladder wall thickening. The WBC was 11.5/μL, total bilirubin was 0.5 mg/dL and CA19.9 was 35 U/mL. Due to high suspicion of a gallbladder mass, a diagnostic laparoscopy was done. Three separate peritoneal nodules as well as a liver nodule were biopsied, all of which were negative on frozen pathology. The surgeon proceeded with open cholecystectomy due to his high suspicion of cancer and frozen section of the gallbladder was negative. There were no masses or abnormal lymph nodes palpated in the gallbladder or liver. Final pathology came back as a T2N0 (stage 2) gallbladder adenocarcinoma. The patient is following up with her surgeon as well as oncology for definitive resection and chemotherapy.

## Discussion

As previously mentioned, gallbladder cancer is a very rare malignancy. As well as rare, the disease has a very high mortality. This is attributable to its lack of early symptomatology leading to advanced stage of presentation and diagnosis in the majority of patients. In areas with the highest incidence, like India and Pakistan, the common denominator among those at highest risk is chronic gallbladder wall inflammation with subsequent cellular proliferation. The most common source of this inflammation is large gallstones greater than 3 cm or chronic Salmonella typhi infection. Although 75-90% of gallbladder cancers had a history of gallstones, only 0.3-3% of cholecystectomies done for presumed benign gallstones showed gallbladder cancer [4, 5].

Few specific risk factors have been identified for gallbladder carcinoma; however, many correlations have been involved with its incidence. These risk factors include large gallstones, female gender, age greater than 65, porcelain gallbladder, gallbladder polyps greater than 10 mm, obesity, chronic infection secondary to salmonella and helicobacter, congenital biliary cysts, anomalous pancreaticobiliary junction, aflatoxin exposure, and diabetes [6-10].

There are four main clinical scenarios a physician may come across when diagnosing a patient with gallbladder cancer. A malignancy may be suspected preoperatively due to clinical presentation. The cancer may be incidentally found on radiologic imaging. Suspicion may arise intraoperatively in a patient undergoing cholecystectomy for presumed benign gallbladder disease, lastly and most commonly, that of incidentally diagnosed malignancy on pathologic examination following a simple cholecystectomy.

In symptomatic patients, pain is the most common presentation followed by anorexia, nausea and/or vomiting. Malaise and weight loss are more ominous symptoms and are suggestive of more advanced disease. In contrast, those patients who present with symptoms more suggestive of acute cholecystitis often have earlier stage disease and a better long-term prognosis. Obstructive jaundice usually indicates direct invasion into the biliary tree and carries a poor prognosis [11, 12].

Like all cancers staging is crucial. The TMN guidelines provide easy survival statistics and treatment guidelines for gallbladder cancer (Table 1).

**Table 1:** TMN Staging of Gallbladder Cancer

T1a	Lamina propia
T1b	Into muscularis
T2	Perimuscular connective tissue
T3	Invading liver or other organ
T4	Invading main portal vein, hepatic artery or two extrahepatic structures
N1	Nodes around the cystic duct, CBD, hepatic artery or portal vein
N2	Aortic, caval, SMA or celiac nodes (not resectable)
M1	Distant metastasis

N1: nodes; N2: aortic, caval, SMA or celiac nodes (not resectable); M1: distant metastasis.

Survival for these cancers is then based on TMN staging, stage 1 being T1 disease with a 5-year survival rate of 50%. Dramatic drop offs in survival are then seen as the disease progresses. The 5-year survival was 29% for stage 2 (T2), 8% for stage 3a (T3), 7% for stage 3b (N1), 3% for stage 4a (T4), and finally 2% for stage 4b (N2 or M1) [13].

Treatment for gallbladder cancer is based on TMN stage. Tis or T1a can be treated with simple cholecystectomy. Any patient with T1b or higher (all four of these patients) will benefit from extended cholecystectomy with 2 cm negative liver margins. This should include a negative margin on the cystic duct; if this margin is involved, there is survival benefit to a CBD resection and hepatico-jejunostomy to uninvolved tissue. All suspicious cases should start with a diagnostic laparoscopy since up to 23% will show disseminated cancer not seen on imaging. If gallbladder cancer is suspected intraoperatively, the procedure should be converted to open to prevent any spillage during the case [14].

Regarding our four patients, the first patient had two risk factors that predisposed her to gallbladder carcinoma. These included female gender and chronic *H. pylori* infection. This patient presented with RUQ pain and back pain for 2 months as well as an unintentional 13 lb weight loss. Ultrasound revealed a distended gallbladder, and CT scan revealed a suspicious gallbladder mass without any evidence of acute cholecystitis. Pathology on this patient came back as adenocarcinoma. Our next patient had five risk factors. These included female gender, age > 65, diabetes mellitus, cholelithiasis, and obesity. This patient presented with abdominal pain for 1 week and was eventually diagnosed with acute cholecystitis. This patient was incidentally found to have adenocarcinoma on pathology. Our third patient had four risk factors including female gender, age > 65, obesity, and cholelithiasis. This patient presented with RUQ pain and was found to have emphysematous cholecystitis with possible gallbladder neoplasm. Our final patient was also a female older than 65 years of age, with cholelithiasis and acute cholecystitis. She presented with epigastric abdominal pain and was found to have a gallbladder mass with irregular gallbladder wall thickening concerning for malignancy. Her pathology came back as gallbladder carcinoma.

In reviewing the literature, the risk factors for gallbladder carcinoma seem to be nonspecific and its presentation may be very difficult to distinguish from more benign pathologies like cholecystitis. The most important underlying risk factors for gallbladder carcinoma are cholelithiasis and female gender. Among our patients, three of four had gallstones. All of our patients were female with three of four being over 65 years of age. Increased BMI has also been correlated with an increased risk for gallbladder cancer. Two of our patients were considered obese by BMI, one was overweight, and one was of normal/healthy BMI. One of our patients had diabetes, two patients presented with acute cholecystitis, and one of our patients had a history of *H. pylori* infection. Given the rare incidence of this disease, four cases presenting over the span of 1 month at a single institution are very unusual. Over the course of 14 years, NBI has only had 21 cases of gallbladder cancer. Imaging via ultrasound and CT scan as well as sending all gallbladders for pathology postoperatively seem to be the best ways to distinguish patients with benign disease from those with malignancy.
